# Transcranial Color Doppler for Assessing Cerebral Venous Outflow in Critically Ill and Surgical Patients

**DOI:** 10.3390/diagnostics16020289

**Published:** 2026-01-16

**Authors:** Amedeo Bianchini, Giovanni Vitale, Gabriele Melegari, Matteo Cescon, Matteo Ravaioli, Elena Zangheri, Maria Francesca Scuppa, Stefano Tigano, Antonio Siniscalchi

**Affiliations:** 1 Postoperative and Abdominal Organ Transplant Intensive Care Unit, IRCCS Azienda Ospedaliero-Universitaria di Bologna, 40138 Bologna, Italy; 2 Internal Medicine Unit for the Treatment of Severe Organ Failure, IRCCS Azienda Ospedaliero-Universitaria di Bologna, 40138 Bologna, Italy; 3 Department of Anesthesia and Intensive Care, Azienda Ospedaliero Universitaria di Modena, Via Pozzo 71, 41125 Modena, Italy; 4 Department of Medical and Surgical Sciences (DIMEC), University of Bologna, 40138 Bologna, Italy; 5 Hepato-Biliary and Transplant Unit, IRCCS Azienda Ospedaliero-Universitaria di Bologna, 40138 Bologna, Italy; 6 Anesthesiology and Pain Therapy, IRCCS Azienda Ospedaliero-Universitaria di Bologna, 40138 Bologna, Italy; 7 Cardiac Surgery Unit, IRCCS Azienda Ospedaliero-Universitaria di Bologna, 40138 Bologna, Italy

**Keywords:** Venous Transcranial Color Doppler, cerebral venous outflow, neuromonitoring, TCCD, TCD, critically ill patients, mechanical ventilation, robotic surgery, cardiovascular disease, intra-abdominal pressure, Trendelenburg position, central venous catheterization, RaCeVa protocol, Internal Jugular Veins

## Abstract

In recent years, Transcranial Color Doppler (TCCD) has gained increasing recognition as a non-invasive neuromonitoring tool. However, there remains a strong tendency to view arterial TCCD as the ‘stethoscope for the brain,’ while the assessment of cerebral venous flow is still underrepresented in clinical protocols. This review aims to explore the emerging role of venous TCCD, particularly when combined with Internal Jugular Vein (IJV) ultrasound, in evaluating cerebral venous outflow in both critically ill and surgical patients. We conducted a narrative review of e-Pub articles from PubMed, MEDLINE, and Scopus, on the pathophysiological factors that impair cerebral venous drainage and their clinical implications in surgical and critical care settings. Based on this evidence, we developed two procedural algorithms that integrate established knowledge of cerebral venous hemodynamics with common clinical conditions affecting venous outflow, including internal jugular central venous catheter placement, mechanical ventilation, and pneumoperitoneum. The algorithms emphasize systematic monitoring of cerebral venous drainage, including assessment of internal jugular vein morphology and Rosenthal’s vein flow, to guide procedural optimization and minimize potential neurological complications. They were informed by validated frameworks, such as the RaCeVa protocol, and are illustrated through two representative clinical case scenarios. Cerebral venous congestion can be induced by multiple established risk factors, including mechanical ventilation, cardiovascular disease, elevated intra-abdominal pressure, the Trendelenburg position, and central venous catheterization. In selected patients, real-time venous TCCD monitoring, combined with IJV assessment, allows early detection of cerebral venous outflow impairment and guides timely hemodynamic and procedural adjustments in both surgical settings and critical care contexts. Venous TCCD neuromonitoring may help prevent intracranial hypertension and its consequent neurological complications. It can guide clinical decisions during procedures that may compromise cerebral venous drainage, such as mechanical ventilation, the placement of large-bore central venous catheters, or laparoscopic and robot-assisted surgeries. Further studies are warranted to validate this strategy and better define its role in specific high-risk clinical scenarios.

## 1. Introduction

Over the past four decades, neurosonology has significantly expanded, becoming an essential and versatile tool in the clinical practice of diverse specialists including neurologists, anesthetists, emergency physicians, and neuro-vascular surgeons. Transcranial Color-Coded Duplex Sonography (TCCD) is an invaluable, non-invasive tool for real-time cerebral hemodynamic assessment in the intensive care unit (ICU). Its diverse applications assist intensivists in diagnosing, monitoring, and guiding therapies for various acute brain injuries and systemic conditions. Key TCCD applications in the ICU include detecting and monitoring cerebral vasospasm, non-invasively estimating intracranial pressure (ICP) (although its accuracy remains modest) and evaluating cerebral autoregulation. TCCD also facilitates real-time detection of microembolic signals (MES) in high-risk patients, monitors cerebral perfusion and vascular recanalization post-stroke, and can support ancillary testing for brain death. However, it is not universally accepted as definitive, as regulations vary by country [1,2,3,4]. Reflecting its clinical relevance, several leading scientific societies have integrated TCCD monitoring into their guidelines [5,6,7,8]. Similarly, TCCD neuromonitoring proves invaluable in the operating room, enhancing surgical outcomes by preventing and managing neurological complications. This includes detecting microemboli release during various surgical procedures (e.g., orthopedic, carotid, and major abdominal surgery) [9,10,11]; non-invasive assessment of ICP, autoregulation and flow velocity during surgery [12,13,14]; and identifying exhausted cerebrovascular reserve during carotid endarterectomy [8,15].

While cerebral arterial flow is routinely monitored in intensive care and surgical settings, cerebral venous outflow assessment remains comparatively neglected. This is evident as the European Society of Intensive Care Medicine’s latest consensus on critical care ultrasonography (ESICM 2021) does not include cerebral venous evaluation among core intensivist competencies [16]. Consistent with this, Goossen et al.’s recent publication on neuromonitoring in mechanically ventilated patients with Acute Brain Injury (ABI) omits ultrasound-based cerebral venous outflow assessment, prioritizing arterial TCCD [17]. This prevalent literary focus mirrors a broader neurocritical care trend, where arterial TCCD is primarily employed for neuromonitoring, often conceptualized as the “stethoscope for the brain.” [18]. Instead, cerebral venous flow assessment remains underrepresented in clinical protocols, despite its pivotal role in intracranial flow hemodynamics and its association with pathologies including intracranial hypertension (IH), venous infarcts, parenchymal hemorrhages, and venous thrombosis [19,20,21,22,23,24,25,26,27].

Factors limiting the integration of venous TCCD into routine clinical practice include the method’s technical limitations (see below) and inconsistent findings regarding the direct correlation between Basal Vein of Rosenthal flow velocities and ICP [24,25,26,27].

Although the direct relationship between cerebral venous outflow and ICP remains debated, cerebral venous flow velocity correlates more strongly with cerebral blood flow (CBF) than with arterial flow velocities [24,25]. This suggests that Basal Vein velocity may serve as a useful indirect indicator of cerebral perfusion. Indeed, decreases in Basal Vein flow velocity have been associated with worse clinical outcomes in patients with subarachnoid hemorrhage complicated by vasospasm and those with traumatic brain injury, thereby highlighting its potential prognostic value [26,27,28].

Collectively, these observations highlight the crucial need for further investigation into the role of cerebral venous flow monitoring in routine neuromonitoring practices, aiming to gain a more comprehensive understanding of intracranial hemodynamics and ultimately improve patient outcomes in both surgical and critical care settings.

Several applications of venous TCCD have been reported in the current literature, including the assessment of cerebral venous thrombosis [22,29], differentiation between vasospasm and hyperemia [30], evaluation of transient global amnesia [31], identification of arteriovenous malformations or fistulas [32], investigation of cerebrospinal venous flow dynamics in Idiopathic IH [20,21], Brain Edema, Midline Shift [24], multiple sclerosis [33], and investigation of neural activation [34]. However, at present, these applications remain primarily confined to specialized environments, such as neurosurgical and neurocritical care settings. With the widespread adoption of Point-of-Care Ultrasound (POCUS) across medical specialities and the increasing availability of high-performance ultrasound devices, cerebral venous flow monitoring has become a feasible and potentially routine practice in various clinical contexts.

## 2. Materials and Methods

This narrative review investigated pathophysiological factors contributing to impaired cerebral venous drainage in both surgical and critical care patients and explored the potential clinical application of venous TCCD neuromonitoring. In these contexts, cerebral venous congestion is commonly induced by established contributors such as mechanical ventilation (MV), cardiovascular disease, elevated intra-abdominal pressure, the Trendelenburg position, and central venous catheter (CVC) placement. In vulnerable patients (e.g., those with ABI), these risk factors can precipitate neurological injury.

Furthermore, to illustrate the potential clinical application of venous TCCD in both surgical and critical care settings, we propose two procedural algorithms and present two representative case scenarios, one from each context, involving patients hospitalized at the IRCCS Azienda Ospedaliero-Universitaria di Bologna, Italy, in 2024.

The proposed algorithms were developed by integrating established pathophysiological knowledge on cerebral venous hemodynamics with clinical conditions known to influence cerebral venous outflow. These conditions, which are commonly encountered in the ICU and operating room, include, for example, internal jugular CVC placement, as well as other high-risk interventions such as MV and pneumoperitoneum.

A central aim of these algorithms is to introduce and emphasize the importance of systematic monitoring of cerebral venous drainage. This encompasses targeted evaluation of internal jugular vein (IJV) morphology and Rosenthal’s vein flow, both before and throughout procedures that may alter intracranial venous dynamics. Such monitoring is intended to support procedural optimization and to guide targeted strategies aimed at minimizing potential adverse neurological effects.

The algorithms additionally draw upon and integrate established, validated frameworks reported in the literature—such as the Rapid Assessment of Cerebral Venous Anatomy (RaCeVa) protocol [35]—thereby situating their conceptual development within a contemporary evidence-based context. Importantly, the algorithms were not generated through the use of Artificial Intelligence and still require validation through randomized controlled trials.

To further illustrate the potential role of venous TCCD monitoring, we present two representative clinical scenarios. These patients exhibited risk factors for impaired cerebral venous return that are frequently observed in the operating room and intensive care setting, including multiple large-bore central venous accesses and obstructive medical conditions such as tamponading pericardial effusion.

From December 2024 to June 2025, we performed a literature search, without publication time limits, including e-Pub published articles in PubMed, MEDLINE, and Scopus, using keywords such as “Venous Transcranial Color Doppler”, “Cerebral Venous Outflow”, “Cerebral Venous Drainage”, “Cerebral Venous Hemodynamics”, “Neuromonitoring”, “Neurocritical Care”, “Intensive Care Unit”, “Intracranial Pressure”, “mechanical ventilation”,” cardiovascular disease”, “intra-abdominal pressure”, “Trendelenburg position”, and “central venous catheterization”. Studies published in English were included, with data extraction focusing on patient population, clinical setting, specific venous TCCD applications, and key findings relevant to the review’s objectives.

Finally, this study was conducted in full compliance with the ethical principles outlined in the World Medical Association’s Declaration of Helsinki and adhered to the guidelines of Good Clinical Practice. Informed consent was obtained from both patients before their participation.

## 3. Results

**Figure 1:**
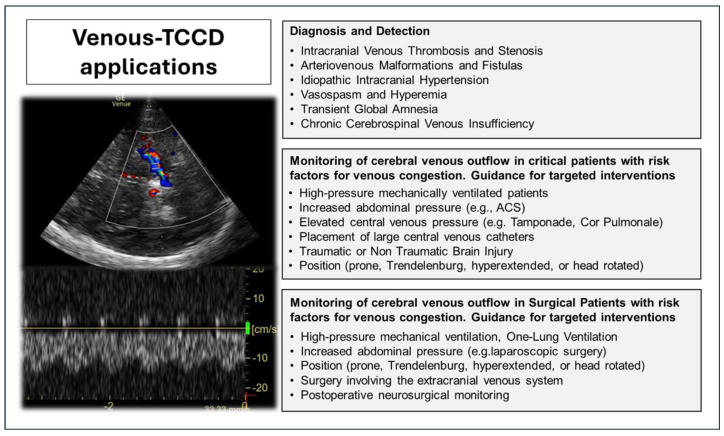
The main potential applications of Venous Transcranial Color-Coded Duplex Sonography (Venous TCCD). ACS—Abdominal Compartment Syndrome.

Figure 1 synthesizes the main potential applications of TCDD. We describe this in detail in the following subparagraphs.

### 3.1. Cerebral Venous Drainage Monitoring in Critical Care Patients

In critically ill patients, various conditions can impair cerebral venous drainage, causing a mismatch between arterial and venous flow and, as a result, leading to IH. These conditions include elevated intrathoracic pressure (e.g., due to MV), increased abdominal pressure (IAP), specific cardiovascular pathologies, and direct venous obstruction [19,20]. Wilson MH et al. and Anirudh Arun et al. proposed classifications for venous outflow obstruction and venous congestion, detailing both their locations and underlying causes. These classifications are considered essential for refining diagnosis and guiding the management of related conditions [19,20].

Below, we review the most common clinical conditions in ICU patients that, individually or in combination, increase the risk of cerebral venous congestion. In these scenarios, assessing cerebral venous flow with venous TCCD may serve as a screening tool, allowing the early identification of impaired intracranial venous outflow before the development of intracranial hypertension detectable through conventional ICP monitoring techniques. By revealing subclinical reductions in venous drainage, venous TCCD can help guide timely, targeted interventions and potentially improve neurological outcomes.

In Supplementary Figure S1, we present a diagnostic and management algorithm for cerebral venous outflow impairment during high-risk procedures and therapies, designed to safeguard neurological health through early detection and effective intervention.

#### 3.1.1. Mechanical Ventilation (MV)

Critically ill patients often require prolonged MV, often requiring high ventilatory pressures and low tidal volumes (TV) [36]. While protective ventilation strategies—employing high positive end-expiratory pressure (PEEP) and low TV—aim to reduce ventilator-induced lung injury, these techniques, together with the prone position used in severe acute respiratory distress syndrome, may modify cerebral venous drainage and influence ICP [19,20,37,38,39]. Hypercapnia resulting from low TV may further increase CBF and worsen ICP, particularly in patients with ABI. Despite the ESICM consensus providing clinical practice recommendations for MV in ABI patients, consistently low GRADE ratings across multiple areas highlight substantial knowledge gaps regarding the feasibility, safety, and efficacy of different management strategies [39].

In this context, assessing cerebral venous flow with venous TCCD can serve as a screening tool, enabling early identification of patients at risk of impaired venous outflow before clinically significant IH develops. This information may help clinicians consider ventilatory strategies within the broader haemodynamic and neurophysiological context [40]. Section 3.3.1 illustrates the effects of MV in a patient with risk factors for cerebral venous congestion. Importantly, reduced venous outflow alone does not dictate modifications to lung-protective ventilation, reflecting the uncertainties in combined lung- and brain-protective management.

#### 3.1.2. Cardiovascular Diseases

Systolic and diastolic heart failure, major valvular diseases, right heart failure, cardiac tamponade, pulmonary hypertension (including secondary forms, e.g., due to obstructive sleep apnoea), and pulmonary embolism are common reasons for ICU admission and can contribute to central venous hypertension and congestion [41,42]. Altered venous pressures may disrupt the balance between cerebral inflow and outflow, potentially affecting ICP [19,20,43,44].

Cerebral venous flow assessment provides additional, real-time information on venous drainage and haemodynamic interactions, helping to identify patients at risk of congestion and guiding the timing and prioritization of interventions in the overall clinical context. While therapies such as fluid management, vasoactive agents, or procedural adjustments may influence venous outflow, their use should be individualized and is not directly determined by venous flow measurements alone. Section 3.3.2 demonstrates how a haemodynamically significant pericardial effusion affects cerebral venous drainage. Venous flow was markedly slowed in both the jugular and axillary veins, presenting as a “smoke effect” (see Supplementary Video S3). The combined evaluation of cerebral venous drainage and focused cardiac ultrasound (FOCUS) allowed rapid identification of the underlying cause and prompt pericardiocentesis.

#### 3.1.3. Increased Abdominal Pressure (IAP)

**Figure 2:**
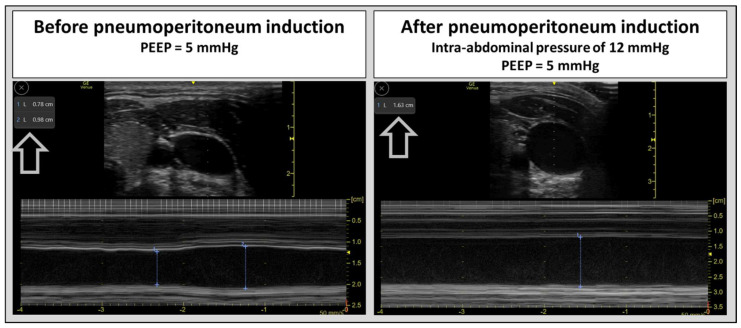
Changes in the internal jugular vein before and immediately after the induction of pneumoperitoneum during laparoscopic surgery (with the same patient position and the same applied probe pressure). The vein appears dilated and exhibits no respiratory fluctuations, indicating increased venous pressure and congestion.

IAP is also common in critically ill patients and is linked to various pathological conditions, including obesity, ascitic decompensation, or abdominal compartment syndrome. IAP raises ICP mainly by blocking cerebral venous outflow. It also contributes to IH through increased intrathoracic pressure, reduced pulmonary compliance (requiring higher PEEP levels), impaired cerebrospinal fluid drainage, and a consequent decrease in cerebral perfusion pressure, especially in those with impaired cerebral autoregulation [19,20,45,46,47]. Incorporating cerebral venous flow assessment into monitoring could help guide targeted interventions, such as paracentesis or laparotomy, to lower abdominal pressures, improve cerebral venous drainage, and thus reduce neurological risks. Figure 2 shows the effects on the structure and the dynamic changes in the IJV after increased iatrogenic abdominal pressure. In this context, hypercapnia resulting from CO_2_ insufflation and compromised pulmonary ventilation during laparoscopy, along with arterial cerebral vasodilation, may worsen the mismatch between arterial and venous cerebral flow, further increasing ICP.

#### 3.1.4. Intracranial or Extracranial Venous Obstructions

Critical patients may sometimes present with intracranial or extracranial venous obstructions. These obstructions can facilitate the propagation of pressure from the neck to the cerebral venous system, due to its valveless structure, potentially causing IH [19,20]. A dramatic rise in ICP occurs in patients with acute venous sinus occlusion [48]. Even extracranial venous flow obstruction, such as in Superior Vena Cava (SVC) Syndrome, IJV compression (for example, from cervical haematoma, cervical masses, or cervical oedema), IJV stenosis, and thrombosis can result in increased ICP [19,20,49,50,51]. Incompetent IJV valves have also been linked with increased ICP and neurological complications [33,52,53]. Monitoring cerebral venous flow in patients with suspected intra- or extracranial venous obstructions can provide complementary information to guide interventions such as thrombolysis or hematoma evacuation. Techniques like blood withdrawal from the sagittal sinus are also described but are uncommon [54].

#### 3.1.5. Centrally Inserted Central Catheters (CICC)

Critically ill patients often require CVC placement, with the most commonly used CICC. These catheters are typically inserted via the jugular or axillary–subclavian vein. The presence of large venous catheters within the IJV can potentially impede venous flow, depending on the size discrepancy between the vein and the catheter [55], as well as the effectiveness of compensatory flows such as those from extra-jugular venous networks, including the spinal epidural and paravertebral venous systems [54]. Other factors, such as periprocedural complications from CICC insertion (e.g., hematoma and thrombosis) and Trendelenburg positioning, can also potentially lead to reduced cerebral venous flow, resulting in congestion and increased ICP [19,20,49]. Unfortunately, the reduced venous drainage caused by CICC has not been extensively investigated. The recent study by Yang B. et al. concluded that IJV cannulation may not be the preferred approach in robot-assisted laparoscopic surgery, as it was identified as a risk factor for IJV venous regurgitation, ICP elevation, and delayed emergence from anaesthesia [53]. In this context, the assessment of intracranial venous flow (e.g., Rosenthal Vein flow) provides valuable information on overall venous drainage, including both the jugular and extrajugular systems. This evaluation can potentially help guide the placement of central vascular access in selected patients, complementing the RaCeVa protocol [35]. In Supplementary Figure S2, we propose an integrated management algorithm combining cerebral venous outflow assessment with the RaCeVa protocol for CICC placement in patients at increased risk of cerebral venous congestion and/or neurological damage (Section 3.3.2 exemplifies the application of the procedural algorithm).

#### 3.1.6. Postural Changes

Posture also plays a vital role in regulating cerebral venous drainage and, consequently, ICP. The Trendelenburg position can affect cerebrospinal fluid dynamics by increasing arterial inflow and reducing venous outflow, potentially leading to elevated ICP, with the magnitude of changes depending on the degree of tilt [19,20,56]. Furthermore, increased intrathoracic pressure, reduced pulmonary compliance (requiring higher PEEP), and elevated central venous pressures can worsen intracranial hypertension. Conversely, several studies have shown the benefits of head elevation and a neutral head position in managing IH. The physiological mechanisms behind this intervention are complex and include improved cerebral venous outflow [54,57,58].

Monitoring cerebral venous flow during positional changes can potentially help verify adequate drainage and optimize patient posture.

### 3.2. Cerebral Venous Drainage Monitoring in Surgical Patients

Like critically ill patients, surgical patients can experience intraoperative changes in cerebral venous drainage, potentially leading to IH.

MV, IAP, Trendelenburg position, and CICC catheterization all affect cerebral venous flow. Furthermore, intraoperative cardiovascular complications (e.g., embolism or right heart failure), jugular sacrifice, and surgeries involving large venous compression can impair cerebral venous outflow and worsen neurological outcomes [51].

An example of surgery combining multiple factors that induce venous congestion is robotic-assisted laparoscopic surgery in gynaecological and urological procedures [59,60]. Here, prolonged Trendelenburg positioning, intra-abdominal insufflation, high PEEP requirements, hypoxic–hypercapnic pulmonary vasoconstriction, air embolism, and CVC placement in the IJV can compromise cerebral venous outflow. Pre-existing cardiovascular or pulmonary conditions that predispose to venous congestion may further exacerbate this impairment. Concurrently, cerebral arterial inflow can increase due to patient positioning, elevated mean arterial pressure, impaired cerebral autoregulation (e.g., with high-dose halogenated anesthetics), and elevated PCO2 levels. This arteriovenous mismatch contributes to elevated ICP and can potentially lead to delayed neurocognitive recovery [53,61,62,63]. Despite these observations, data on the association between robot-assisted surgery and postoperative neurocognitive dysfunction are inconsistent [64,65]. Some authors recommend preventive strategies, including limiting the duration of steep Trendelenburg positioning, restricting fluid intake, minimizing operative time, and reducing intra-abdominal insufflation pressure to 8 mmHg [66].

Intraoperative monitoring of cerebral venous flow could enable the early detection of venous congestion and guide strategies to minimize neurological damage. Figure 2 illustrates changes in the IJV before and immediately after pneumoperitoneum induction during laparoscopic surgery (12 mmHg). The vein appears dilated and exhibits no respiratory fluctuations, indicating increased venous pressure and congestion.

### 3.3. Clinical Utility of Venous TCCD: Representative Cases from Surgical and Critical Care Settings

To better illustrate the potential role of venous TCCD monitoring in both intensive care and surgical settings, we present two representative case scenarios, one from each context. These patients exhibited risk factors for impaired cerebral venous return, such as liver transplantation (LT) procedure and hemodynamically significant pericardial effusion, conditions that may contribute to elevated ICP and subsequent neurological injury. LT is a complex procedure often associated with neurological complications like cerebral oedema. In LT patients, neuroinflammation linked to impaired cerebral venous return can worsen neurological outcomes. This impairment is driven by factors such as MV, the placement of multiple and large CICC (Figure 2), and elevated post-reperfusion right atrial pressure (resulting from right ventricular dysfunction, fluid overload, and pulmonary embolism). ICP is commonly assessed in patients undergoing LT using indirect, non-invasive techniques, such as the measurement of the optic nerve sheath diameter (ONSD) [14]. Pericardial effusion is another condition that can elevate IJV pressure (a component of Beck’s Triad) by impeding right ventricular filling, potentially diminishing cerebral venous drainage [20]. The Rosenthal Vein, assessed alongside the IJVs, shows promise for comprehensive and rapid evaluation of overall cerebral venous flow, including both jugular and extrajugular components.

Here, we describe the Rosenthal Vein Sampling Technique:

According to published neurosonological and neuroimaging Doppler literature, insonation of the Rosenthal Vein is feasible via the transtemporal acoustic window by targeting deep venous structures in the mesencephalic–diencephalic region [67]. In our study, the Rosenthal Vein was assessed using a sector transducer optimized for low-flow venous signals, with a low pulse repetition frequency, low wall filters, and high gain to enhance sensitivity to slow venous velocities. Axial (transverse) scanning was performed in the superior mesencephalic/diencephalic plane, where deep venous structures are typically visualized.

Only one Rosenthal Vein was insonated, as the two veins are physiologically interconnected and drain into the same deep venous system. The primary anatomical landmark for identifying the Rosenthal Vein is the P2 segment of the posterior cerebral artery (PCA), which serves as a reliable reference structure because of its consistent location and orientation. Once the ipsilateral PCA-P2 tract was identified, the probe was tilted slightly, and fine adjustments in depth and angle were made until the Rosenthal Vein appeared posterior and somewhat inferior to the artery. As described in the literature, the Rosenthal Vein typically exhibits flow directed away from the probe, providing a distinct spectral Doppler signature (as shown in Video S1). The study of Valdueza et al. [67] demonstrated the feasibility of using transcranial Doppler ultrasound to assess deep cerebral veins, specifically the basal vein of Rosenthal, which was identified by its anatomical relationship to the PCA and flow direction. Normal mean blood flow velocity ranged from 4 to 17 cm/s, with a mean of 10.1 ± 2.3 cm/s, showed no significant side-to-side differences, and decreased with age, primarily in men aged 40 years or older.

#### 3.3.1. Case 1

**Figure 3:**
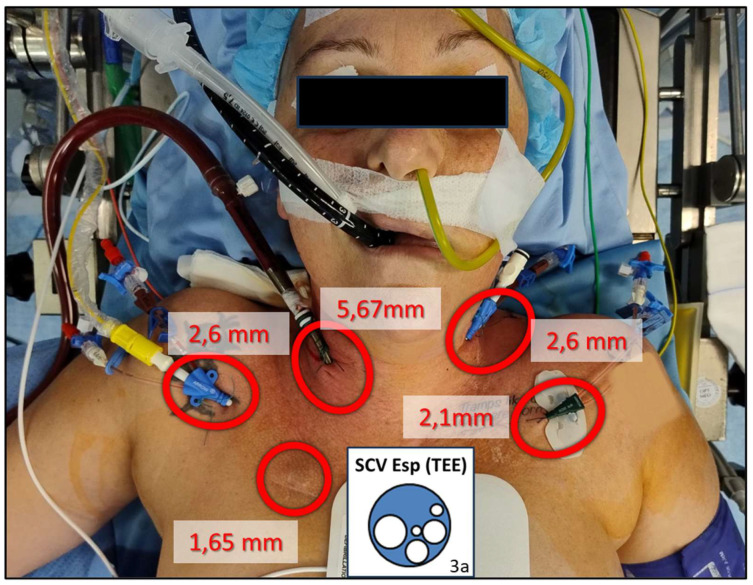
A 50-year-old patient (Section 3.3.1) underwent liver transplantation due to hepatocellular carcinoma. The procedure was performed with a veno-venous bypass. Multiple vascular devices were positioned in the cervico-thoracic region. 3a illustrates the relationship between the diameter of the Superior Cava Vein (SCV) measured using transesophageal echocardiography (TEE) during expiration (Esp) and the external diameter of the vascular devices.

Here, we present an example of Rosenthal Vein flow assessment in a patient scheduled for LT with veno-venous bypass. The patient required large-bore central venous catheters in both the IJV and axillary veins, with a significant reduction in the lumen of the SVC (see Figure 3).

**Figure 4:**
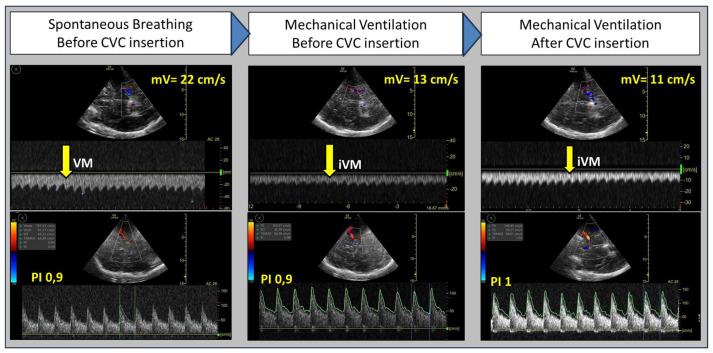
Flow of the Rosenthal Vein and the Middle Cerebral Artery (MCA) in a patient scheduled for liver transplantation (Section 3.3.1). The evaluation was performed at three successive time points: before general anesthesia, after tracheal intubation and initiation of Mechanical Ventilation (MV), and after the positioning of vascular devices. While the MCA Pulsatility Index (PI) remained nearly stable across the three time points, venous flow was significantly reduced by MV (PEEP at 5 cm H_2_O) and the presence of vascular catheters. Additionally, venous flow failed to increase with either spontaneous or mechanically induced Valsalva Maneuver (30 cm H_2_O for 15 s, as described by Tiecks et al. [68]) after vascular device placement. VM = Valsalva Maneuver, iVM = induced Valsalva Maneuver, mV = mean Velocity in Rosenthal Vein.

As depicted in Figure 4, MV (PEEP of 5 cm H_2_O) and the presence of vascular catheters significantly reduced cerebral vein flow without changes in systemic arterial pressure and arterial PCO_2_.

Furthermore, post-device placement, an additional diminution in cerebral venous flow velocity was noted during the Valsalva Maneuver (VM) dynamic load test. This finding suggests that the venous system was likely incapable of accommodating the heightened venous blood flow, considering that under physiological conditions of cerebral venous drainage, VM is associated with an increase in Rosenthal Vein (phase IV) [68,69].

Despite the decrease in venous flow, no simultaneous increase in ONSD and cerebral arterial resistance was observed (as indicated by the pulsatility index—PI). This suggests that alterations in venous flow occurred earlier than changes in other non-invasive parameters used for ICP estimation. This aspect holds significant clinical importance, as the duration and intensity of IH episodes are independent predictors of mortality and severe disability in several studies, underscoring the need for prompt diagnosis to improve patient outcomes [70].

**Figure 5:**
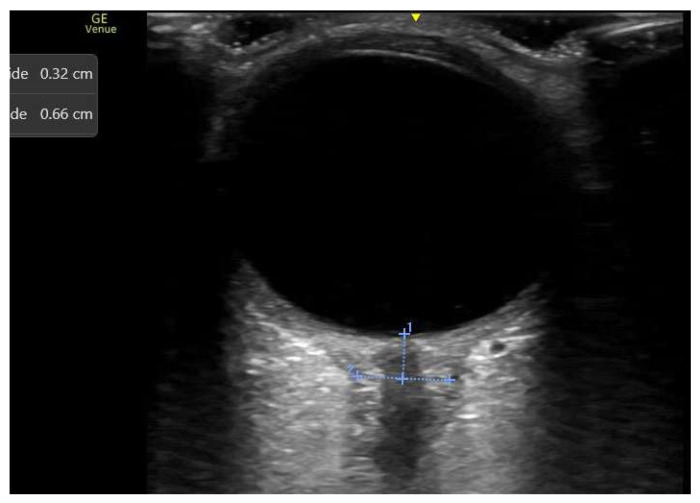
Optic Nerve Sheath Diameter (ONSD) at the end of the liver transplant surgical procedure (Section 3.3.1). The optic nerve sheath appears dilated due to increased intracranial pressure.

Based on this assessment, several intraoperative measures were implemented to reduce the risk of IH caused by decreased venous outflow. These included serial monitoring of cerebral flows and ONSD, prompt intraoperative removal of unnecessary vascular devices, use of low PEEP levels, head positioning, anti-Trendelenburg positioning, PCO_2_ regulation, and careful management of fluids and coagulation. After the approximately 8-h surgery, bilateral ONSD were dilated compared to preoperative values (6.6 mm vs. 4.5 mm) and appeared tortuous, indicating an increase in ICP (Figure 5, Supplementary Video S2). On the first postoperative day, the patient experienced mild headache and refractory vomiting despite antiemetic therapy, with no other neurological signs. Neurological symptoms had resolved by the second postoperative day, and ONSD returned to its preoperative level.

#### 3.3.2. Case 2

A 72-year-old patient with sepsis secondary to pneumonia required CVC placement. The patient was febrile, mildly disoriented, slightly tachypneic, oliguric, with a mean arterial pressure of 60 mmHg, and a history of resolved pericarditis.

**Figure 6:**
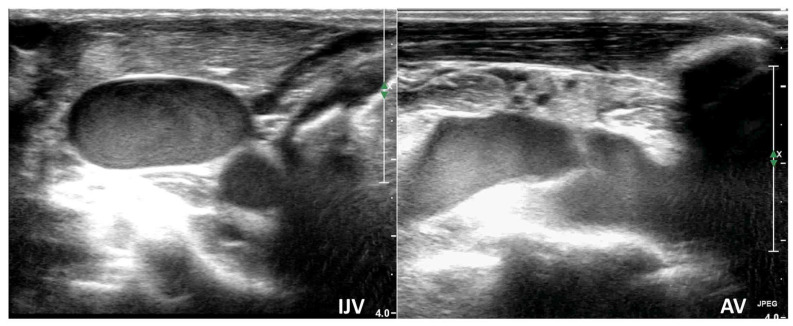
The jugular and axillary veins appeared dilated, with absent respiratory variations and a “smoke effect” flow pattern in a patient (Section 3.3.2) with hemodynamically significant unrecognized pericardial effusion (see Supplementary Video S3). AV: Axillary Vein; IJV: Internal Jugular Veins.

During the RaCeVa protocol, the jugular and axillary veins appeared dilated, with a “smoke effect” and absent respiratory variations (Supplementary Video S3, Figure 6). Rosenthal Vein flow could not be sampled due to very low velocity, suggesting impaired cerebral venous outflow and prompting focused ultrasound evaluation.

FOCUS cardiac ultrasound identified a hemodynamically significant circumferential pericardial effusion, with early effects on right heart chamber filling and a distended inferior vena cava, confirming the need for urgent pericardiocentesis. A temporary femoral CVC was placed prior to pericardiocentesis. Mechanical ventilation and diuretics were withheld until oxygenation remained adequate. Neurological and haemodynamic status improved after drainage. This case illustrates the application of the procedural algorithm in Supplementary Figure S2, highlighting the role of cerebral venous ultrasound as a screening tool to detect venous congestion and provide complementary information for clinical decision-making, particularly when congestion is not clinically apparent.

### 3.4. Limitations of Venous TCCD in Clinical Practice

While venous TCCD shows promise for routinely assessing cerebral venous outflow, several limitations currently hinder its widespread clinical use. First, poor acoustic windows are a major obstacle in many patients, especially the elderly or those with thick temporal bones. This reduces image quality and the accuracy of flow measurements. Second, high-performance ultrasound systems with specialised software are not widely available, particularly in resource-limited settings. The technique is also highly operator-dependent, requiring significant expertise in cerebral venous anatomy, hemodynamics, and specific insonation methods. Furthermore, patient access can be limited in environments such as the perioperative setting or ICU due to surgical dressings, monitoring devices, or craniotomy sites, which make examinations challenging. Another important limitation is the absence of standardised reference values for intracranial venous flow velocities, complicating quantitative interpretation. This is probably because flow velocities are affected by many systemic and local factors, including systemic blood pressure, cerebral autoregulation, patient positioning, intrathoracic and intra-abdominal pressure changes, anemia, cerebral hyperaemia, vasospasm, and arteriovenous malformations or fistulas. In this context, evaluating the variation in venous flow velocity may be more clinically relevant than its absolute value (as seen in Section 3.3.1).

## 4. Conclusions

In conclusion, venous TCCD emerges as a promising yet currently underutilized, tool for optimizing cerebral hemodynamics. Its ability to provide insights into impaired cerebral venous outflow suggests that venous TCCD may detect early signs of venous congestion before the development of secondary IH and changes detectable through arterial TCCD or ONSD assessment. This could make venous TCCD a non-invasive method for predicting, preventing, and managing cerebral venous congestion and related neurological complications in both critically ill and surgical patients.

Integrating venous TCCD into neuromonitoring protocols can help guide procedures that risk increasing ICP and support a range of targeted interventions, such as adjusting ventilatory parameters, carrying out cardiovascular therapies or procedures, applying abdominal decompression strategies, and making postural modifications. To assist with this, we have introduced integrated neuro-POCUS algorithms for evaluating cerebral venous outflow in patients undergoing procedures that might impair venous flow drainage.

Future studies are essential to assess further cerebral venous flow alterations and ICP in diverse clinical scenarios, validating the accuracy of the proposed algorithms and paving the way for wider clinical adoption.

### Key Messages

1. Cerebral venous outflow plays a pivotal role in intracranial hemodynamics and is implicated in IH and neurological complications.
2. Despite the widespread use of arterial Transcranial Color Doppler (TCCD) for neuromonitoring, the assessment of cerebral venous outflow remains largely underrepresented in current clinical protocols.
3. Venous TCCD, combined with IJV ultrasound, offers real-time, early detection of cerebral venous outflow impairment, allowing timely and targeted adjustments to prevent secondary IH in critically ill and surgical patients.
4. Integrated venous TCCD offers a novel approach to guide interventions (e.g., ventilation, posture) in patients at risk of impaired cerebral venous outflow. Further studies are needed for broader validation and adoption.

## Data Availability

The original contributions presented in this study are included in the article/Supplementary Materials. Further inquiries can be directed to the corresponding author.

## Supplementary Materials

The following supporting information can be downloaded at: https://www.mdpi.com/article/10.3390/diagnostics16020289/s1, Video S1: Transcranial color Doppler imaging of the Rosenthal Vein and its confluence with the vein of Galen; Video S2: Elevated Intracranial Pressure indicated by bilateral dilation and optic nerve sheath diameter tortuosity post-surgery; Video S3: The jugular and axillary veins appeared dilated, with absent respiratory variations and a “smoke effect” flow pattern; Figure S1: A Proposed Diagnostic and Management Algorithm for Cerebral Venous Outflow Compromise During High-Risk Procedures: Safeguarding Neurological Outcomes Through Early Detection and Management of Compromised Cerebral Venous Drainage; Figure S2: A proposed Extended RACEVA Protocol: Assessment of Cerebral Venous Drainage in Patients Requiring an IJV CICC at Increased Risk of Developing Cerebral Venous Congestion and/or Neurological Damage.

## Abbreviations

ABI	Acute Brain Injury
ACS	Abdominal Compartment Syndrome
CBF	Cerebral Blood Flow
CICC	Centrally Inserted Central Catheters
CVC	Central Venous Catheter
ESICM	European Society of Intensive Care Medicine
ESP	Expiration
FOCUS	Focused Cardiac Ultrasound
IAP	Increased Abdominal Pressure
IH	Intracranial Hypertension
ICP	Intracranial Pressure
ICU	Intensive Care Unit
IJV	Internal Jugular Vein
iVM	Induced Valsalva Maneuver
LT	Liver Transplantation
MCA	Middle Cerebral Artery
MES	Microembolic Signals
MV	Mechanical Ventilation
mV	mean Velocity in Rosenthal Vein
ONSD	Optic Nerve Sheath Diameter
PCA	Posterior Cerebral Artery
PEEP	Positive End-Expiratory Pressure
PI	Pulsatility Index
POCUS	Point of Care UltrasoundRaCeVa
RaCeVa	Rapid Assessment of Cerebral Venous Anatomy
SVC	Superior Vena Cava
TCCD	Transcranial Color Doppler
TCD	Transcranial Doppler
TEE	Transesophageal Echocardiography
TV	Tidal Volume
VM	Valsalva Maneuver
