# Supplementary material for: Transcranial Color Doppler for Assessing Cerebral Venous Outflow in Critically Ill and Surgical Patients
Source: Diagnostics (Basel). 2026 Jan 16;16(2):289. doi: 10.3390/diagnostics16020289 (PMC12840142; doi:10.3390/diagnostics16020289)
Supplement: Supplementary file 1 [file diagnostics-16-00289-s001.zip › Figures S1 and S2.pdf]

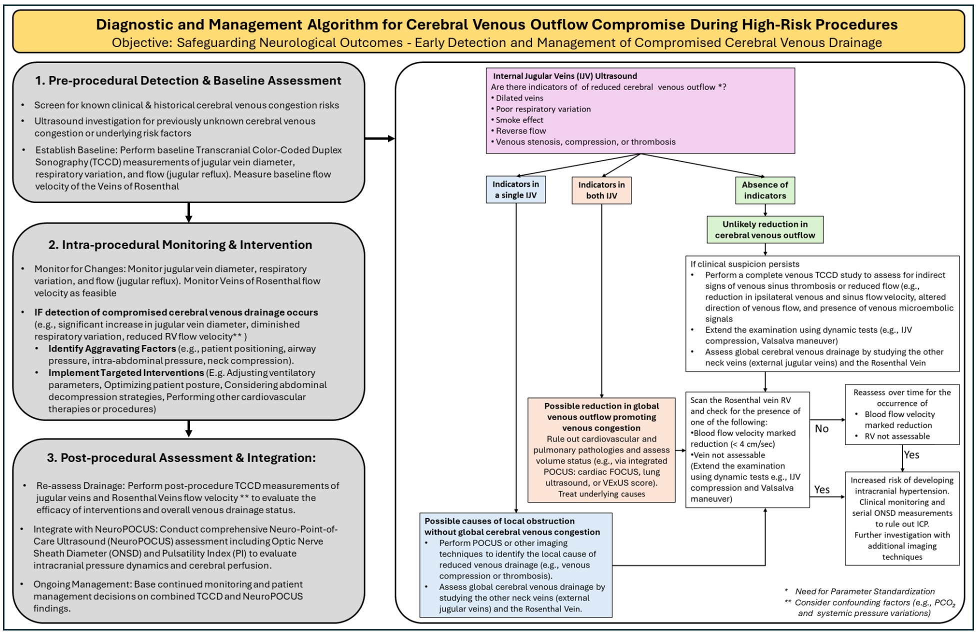

Figure S1. A Proposed Diagnostic and Management Algorithm for Cerebral Venous Outflow Compromise During High-Risk Procedures: Safeguarding Neurological Outcomes Through Early Detection and Management of Compromised Cerebral Venous Drainage. This algorithm serves as a screening tool for patients undergoing procedures with the potential to impair cerebral venous outflow, such as robotic surgery in extreme Trendelenburg position. Its primary objective is to identify unknown risk factors (like right ventricular dysfunction or pericardial effusion) that, when combined with the procedural impact, could lead to cerebral venous congestion. A secondary objective is to promptly monitor and correct any emerging cerebral venous drainage compromise. The algorithm integrates the evaluation of the Internal Jugular Veins (IJV), transcranial color-coded Doppler (TCCD) of venous structures, and Point-of-Care Ultrasound (POCUS), including Focused Cardiac Ultrasound (FOCUS), Venous Excess Ultrasound Score (VExUS), and Optic Nerve Sheath Diameter (ONSD). A limitation of the algorithm is the lack of standardized reference values (*). The 4 cm/s lower velocity limit for the Basal Vein of Rosenthal was derived from the work of Valdueza J et al. [67].

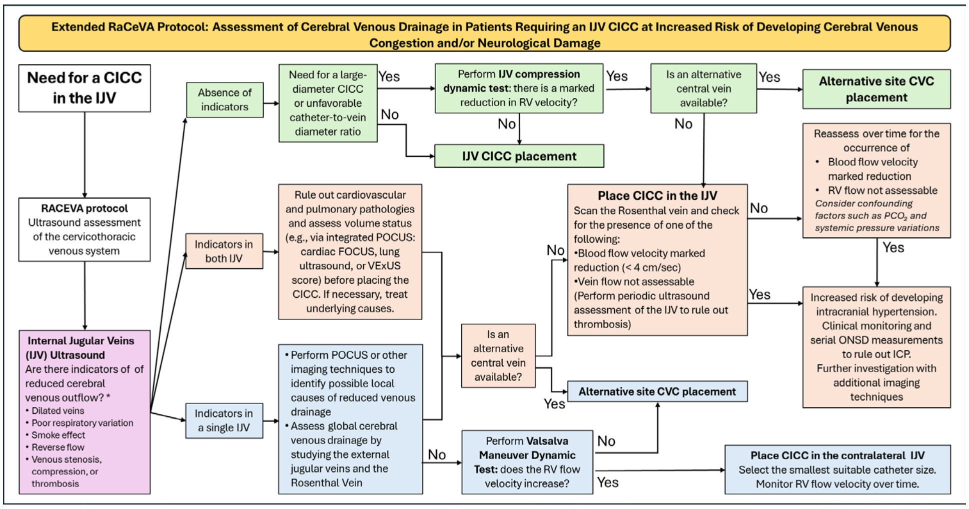

Figure S2. A proposed Extended RACEVA Protocol: Assessment of Cerebral Venous Drainage in Patients Requiring an IJV CICC at Increased Risk of Developing Cerebral Venous Congestion and/or Neurological Damage. Central Inserted Central Catheter: CICC; RV: Rosenthal Vein; FOCUS: Focused Cardiac Ultrasound; POCUS: Point-of-Care Ultrasound; VExUS: Ve-nous Excess Ultrasound Score; ONSD: Optic Nerve Sheath Diameter; ICP: Intracranial Pressure; CVC: Central Venous Catheter; RaCeVa: Rapid Assessment of Cerebral

Venous Anatomy. A limitation of the algorithm is the lack of standardized reference values (*). The 4 cm/s lower velocity limit for the Basal Vein of Rosenthal was derived from the work of Valdueza J et al. [67].
